# Community case management of chest indrawing pneumonia in children aged 2 to 59 months by community health workers: study protocol for a multi-country cluster randomized open label non-inferiority trial

**DOI:** 10.18203/2349-3259.ijct20201719

**Published:** 2020

**Authors:** Enhanced Management of Pneumonia in Community (EMPIC) Study Group

**Keywords:** Children under five years of age, Chest indrawing pneumonia, Enhanced community case management, iCCM, Community level health worker, Amoxicillin, Pulse oximeter

## Abstract

**Background:**

The World Health Organization (WHO) integrated management of childhood illness (IMCI) protocol recommends treatment of chest indrawing in 2–59 months old children with oral amoxicillin by trained health facility workers. Whereas, the WHO/UNICEF integrated community case management (iCCM) protocol recommends referral by community level health workers (CLHWs) to a health facility. This study aims to evaluate whether CLHWs can treat chest indrawing pneumonia effectively and safely.

**Methods:**

This multi-centre cluster randomized controlled open label, non-inferiority trial will be conducted in Bangladesh, Ethiopia, India and Malawi. All sites will use a common protocol with the same study design, participants, intervention, control and outcomes. CLHWs will identify 2–59 months old children with chest indrawing. Study supervisors, trained in the iCCM protocol, will confirm CLHWs’ findings. Pulse oximetry will be used to identify hypoxaemic children. In the intervention group, enrolled children will be treated with oral amoxicillin for 5 days, and in the control group they will be referred to a health facility, after providing first dose of oral amoxicillin. An independent outcome assessor will visit each enrolled child on days 6 and 14 of enrolment, to assess study outcomes.

**Conclusions:**

If CLHWs can effectively and safely treat chest indrawing pneumonia in 2–59 months old children, it will increase access to pneumonia treatment substantially, as in many settings, health facilities and trained health workers are not easily accessible. Moreover, this evidence will contribute towards the review of the current iCCM protocol and its harmonization with the IMCI protocol.

**Trial Registration:**

The trial is registered at AZNCTR International Trial Registry as ACTRN12617000857303.

## INTRODUCTION

Of an estimated 0.8 million pneumonia deaths in children under-five years, majority occur in sub-Saharan Africa and South Asia.^1^ Progress in these regions is slow and the disparity between Africa and the rest of the world has grown.^2,3^ As many as 138 million pneumonia episodes are estimated to occur every year.^4^ Mortality among children with pneumonia varies between 0.1% and 30%, depending upon age, severity of signs, promptness and appropriateness of treatment.^5–8^

The World Health Organization (WHO)/UNICEF integrated Community Case Management (iCCM) protocol recommends that community level health workers (CLHWs) refer all children under five years of age with lower chest indrawing to a referral health facility/hospital.^9^ However, many families do not accept referral advice for a variety of reasons, thus reducing access to treatment.^3,10,11^ Several studies reported effectiveness of oral amoxicillin for chest indrawing pneumonia in 2–59 months old children.^12–16^ WHO revised pneumonia treatment guidelines recommending oral amoxicillin for treatment of chest indrawing pneumonia in 2–59 months old children by trained health workers on outpatient basis.^17^ The 2014 Integrated Management of Childhood Illness (IMCI) chart booklet for health facilities is based on this recommendation, which was not extended to the iCCM protocol for community because of insufficient safety and efficacy evidence.^18^

Access to pneumonia treatment could increase substantially if evidence were generated to show effectiveness and safety of oral amoxicillin for chest indrawing pneumonia in 2–59 months old children treated by CLHWs at community level. Additionally, it could decrease referrals, thereby reducing the load on overburdened hospitals and diminishing treatment costs. The overall goal of this study is to contribute to the evidence base for potential revision of the iCCM protocol and its harmonisation with the IMCI protocol.

### Objectives

The primary objective of this study is to determine and compare treatment failure rates of enhanced iCCM compared with standard iCCM in children aged 2–59 months of age with chest indrawing pneumonia without general danger signs and/or hypoxaemia.

The secondary objective is to evaluate the use of pulse oximetry and oxygen saturation (SpO_2_) as a component of a community-based pneumonia management algorithm; specifically, the feasibility of using pulse oximetry by CLHWs and its acceptability by them and mothers/caregivers of children.

## METHODS

### Study design, setting and participants

We designed a cluster randomized controlled trial (CRCT) to be conducted in two African (Ethiopia and Malawi) and two Asian sites (Bangladesh and India). The key characteristics of sites of this open-label, non-inferiority CRCT are shown in Table 1 and explained elsewhere.^19^

### Participants

Children aged 2–59 months with chest indrawing pneumonia whose parents or caregivers consent for participation will be enrolled in the study. Children will be excluded if they have current history of cough for 14 days or more, diarrhoea for 14 days or more, blood in stools, fever for seven days or more, convulsions, persistent vomiting, oedema of both feet, hypoxaemia (SpO_2_ <90%), are unusually sleepy or unconscious on observation, severely malnourished - mid-upper arm circumference (MUAC) <11.5 cm (for 6 months and above age), or were previously enrolled in study.

### Sample size

If there is truly no difference between the standard iCCM and enhanced iCCM (assuming 10% treatment failure in both groups), with design effect of 1.6 and attrition of 10%, then 1340 children 2–59 months of age with chest indrawing per group are required to be 90% sure that the upper limit of a two-sided 95% confidence interval will exclude a difference in favour of the standard iCCM of >5%. Assuming a pneumonia incidence of 0.25 episodes per child per year, and 15% having chest indrawing pneumonia, 30 cases are expected to be enrolled per cluster over the study period of two years. In order to obtain this number, 90 clusters (45 clusters per group) are required. A parallel trial in young infant up to 2 months with fast breathing pneumonia will be carried out at the same study sites and at the same time, which requires 198 clusters (99 clusters in each arm).^19^ We chose the same number of clusters for this trial of children 2–59 months of age with chest indrawing, even though only 90 clusters (45 in each group) were required.

### Study intervention

In the intervention group, all enrolled children with lower chest indrawing will be treated with 80 mg/kg/day amoxicillin in two equal doses for five days. Oral dispersible amoxicillin 250 mg scored tablets will be used, which will be dissolved in expressed breast milk for exclusively breastfed infants. CLHWs will administer the first dose of amoxicillin DT at the place of enrolment and the remaining doses will be provided to mothers or caregivers for administering at home. Mothers/caregivers will be counselled on dose frequency, duration of therapy and importance of adherence. A summary of intervention is shown in Table 2.

### Control

In the control group, CLHWs will refer all children with chest indrawing pneumonia to the nearest health facility in Malawi and to a higher level health facility in India, Bangladesh and Ethiopia.

### Study outcomes

#### Primary outcomes:

Treatment failure will be defined as death at any time within day 1 to day 14 of enrolment, hospitalized for any reason or with any indication for hospitalization on day 6 of enrolment, persistence of chest indrawing on day 6 of enrolment, development of serious adverse effect (anaphylactic reaction, severe diarrhoea, disseminated and severe rash) due to amoxicillin within day 1 to 5 of enrolment.

Treatment failure will only be assessed and declared by an independent outcome assessor (IOA). Withdrawal of informed consent at any time after admission in the study, as well as hospitalization decided by the family, will be recorded but will not be considered as treatment failure.

#### Secondary outcomes:

Feasibility of using a pulse oximeter as part of iCCM; accuracy of pulse oximetry when used by CLHWs against a standardized measurement by a trained supervisor; and impact of pulse oximetry on referral and treatment outcomes i.e., does it increase safety of iCCM and improve referrals?

### Study procedures

#### Screening and enrolment

**Figure 1:**
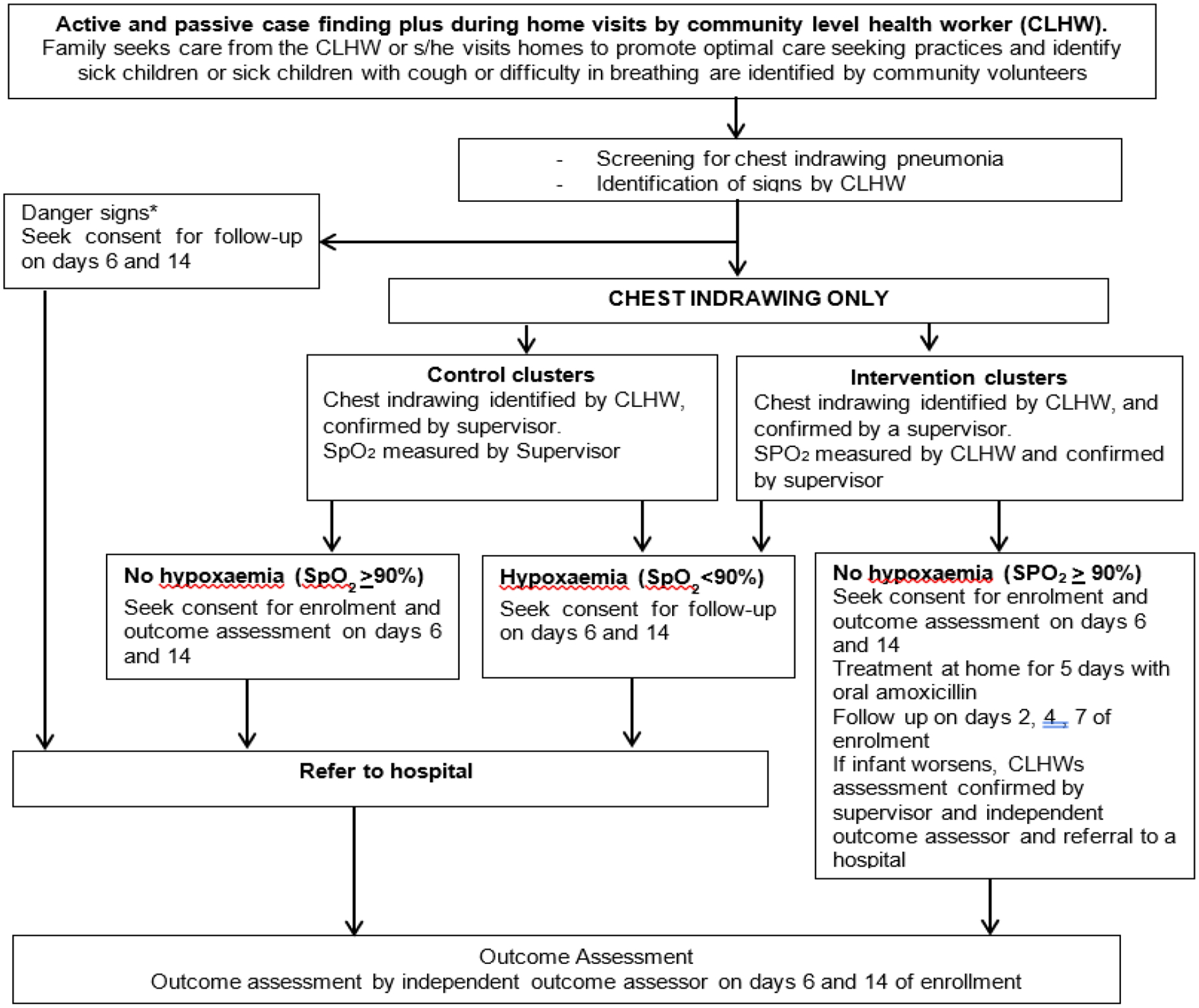
Overall study approach for screening, enrolment and management of sick children. *Danger signs - cough for 14 days or more, diarrhoea for 14 days or more, blood in stools, fever (temperature equal to above 38°C/100.4°F) for seven days or more, convulsions, persistent vomiting (defined as vomiting following three attempts to feed the baby within ½ hour), unusually sleepy or unconscious, severely malnourished as identified through mid-upper arm circumference [MUAC] < 11.5 cm (for 6 – 59 months) or swelling of both feet.

Children aged 2–59 months presenting to a CLHW with cough and/or difficult breathing will be evaluated using eligibility criteria for enrolment (Figure 1). As the CLHWs in Bangladesh and Ethiopia are based at a small health facility and cover a larger population and geographical area compared to the CLHWs in India and Malawi who are based at community level, community volunteers in Bangladesh and Ethiopia will be trained to make home visits to help identify sick children and link them to the CLHWs.

In the intervention group, the CLHWs will take a detailed patient history, perform clinical examination and record the findings on a standardized case recording form (CRF). The CLHWs will also perform pulse oximetry on all children with chest indrawing to identify hypoxaemia (SpO_2_ <90% by pulse oximetry). Children having any danger sign or hypoxaemia, or whose mothers/caregivers don’t consent to participate in the study will be referred to a referral level health facility after the first dose of amoxicillin. Parents/caregivers of these children will be requested to give consent for follow-up by an IOA on days 6 and 14. In the control group, these children will be referred to a referral level health facility. A detailed patient history and clinical examination will be performed by the CLHW, and findings will be recorded on a standardized CRF.

All children meeting the eligibility criteria will be examined by a study supervisor to validate the CLHW’s findings in both intervention and control groups. Those fulfilling the eligibility criteria will be enrolled after a written informed consent is obtained from the mother or caregiver. Screening, clinical assessment, enrolment and provision of study medication will take place within one hour of identifying the case. Details of the implementation strategy at various sites are summarized in Table 3.

### Randomization, allocation and masking

The cluster or unit of randomization is Union in Bangladesh, Health Sub-center in India, and Health Center in Ethiopia and Malawi. Stratified randomization has been carried out based on the population of the clusters i.e., clusters divided into two stratums, median or above and below the median. Randomization list has been prepared by a WHO staff not involved with the site work. For Bangladesh, 52 clusters (26 in intervention and 26 in control), in Ethiopia 20 clusters (10 in intervention and 10 in control), in India 92 clusters (46 in intervention and 46 in control) and in Malawi, 44 clusters (22 in intervention and 22 in control) were selected. Masking of participant and study team is not possible due to nature of the intervention. However, attempts will be made to keep the outcome assessors unaware of group allocation.

### Use of pulse oximetry

Hypoxaemia is a major contributor to mortality and a major complication of pneumonia,^20–23^ but is difficult to identify clinically. Supplemental oxygen needed for management of hypoxaemic children is neither consistently available in low- and middle-income countries nor always appropriately used.^23–26^ Using non-invasive pulse oximetry technology, hypoxaemic children at a higher risk of poor outcomes can be identified and promptly referred to a health facility that has oxygen. Current iCCM tool does not identify hypoxaemic children. CLHWs will be trained to use pulse oximetry in the intervention group. This will increase the safety of study participants and improve referrals. *Masimo* RAD-5v handheld portable pulse oximeters will be provided to CLHWs, study supervisors and independent outcome assessors in intervention clusters, but only to study supervisors and independent outcome assessors in the control group. They will be trained to use and maintain them.

### Follow-up and assessment of adherence to treatment

In the intervention group, CLHWs will follow all enrolled children on days 2, 4 and 7. The mothers/caregivers will be advised to return to the community clinic in Bangladesh, health post in Ethiopia, and to a CLHW in India and Malawi for scheduled follow-ups. Mothers/caregivers will be explained all danger signs and counseled on when to contact CLHWs any time between the scheduled follow-up visits. CLHW will visit a child at home who does not come for follow-up.

During the follow-up visits, CLHWs will evaluate the child for clinical signs. If any danger sign or deterioration is observed, the CLHW will call the study supervisor to physically confirm the findings and refer the child to a higher-level health facility as per iCCM protocol. CLHWs will check for presence of adverse drug reactions during follow up visits. They will count the remaining tablets to assess adherence. In instances when follow-up visit is scheduled after the completion of treatment or the parents/caregivers lose/misplace the remaining medicine, caregiver will be asked about the frequency of administration and the number of tablets used. An enrolled child will be considered adherent if s/he has received either all oral amoxicillin doses for five days or at least 8 doses (80%). Adherence to treatment provided to the children in the control group will also be assessed in the same manner.

The mobile phone numbers of the CLHWs and study supervisors will be shared with mothers/caregivers to contact them if any adverse event occurs between the follow-up visits.

### Outcome ascertainment

IOA will visit enrollees in both groups on days 6 and 14 of enrolment, at home or in hospital, to assess outcomes. The IOA will clinically examine the child and perform pulse oximetry.

### Separation between the outcome assessment and treatment teams

The proposed trial cannot be blinded due to the nature of the treatment, so we have incorporated independent outcome assessment to reduce potential measurement bias. We will hire trained nurses, physicians or clinical field workers as IOA who will be independent of the enrolment and treatment to evaluate study outcomes. IOAs will be trained specifically for research purposes in the standard iCCM, enhanced iCCM and study procedures.

### Demand generation activities

Families will be encouraged to seek care from CLHWs for their sick children through extensive advocacy and information, education, and communication (IEC) activities. In the intervention group, IEC activities will aim to create awareness about the CLHWs’ new role to provide treatment for chest indrawing pneumonia. In the control group, the IEC activities will focus on sustaining the community’s demand for standard iCCM treatments provided by CLHWs. The methods will include posters with pictures and simple messages in local languages, and audio announcements. Posters and wall paintings will be placed at high-visibility public places i.e., government buildings, marketplaces and village meeting venue.. The local CLHW’s name and phone number will be pasted outside each CLHW’s home in India and Malawi so that the community can contact them easily. Periodic loudspeaker announcements will be made across the study areas. Governments use these methods to promote new programmes.

Members of existing community support groups will be sensitized about the signs of illness in children to disseminate them. Parents or caregivers will be shown video clips on chest indrawing at immunization centers and mothers’ gatherings at primary schools. Pictorial leaflets will be distributed, and discussion sessions will be held in schools on pneumonia features.

### Quality control and assurance

#### Training and standardization of CLHWS and study teams

The training of CLHWs, study supervisors, IOAs, study coordinators and investigators will be a two-step process. WHO experts will facilitate a six day training course for master trainers which, will be organized for selected senior level participants at one of the sites using the study standard operating procedures, and the WHO modules on “Caring for the Sick Child in the Community module” and pulse oximetry.^27^ These master trainers will then train the other study staff at their respective sites. For the intervention group the iCCM chart booklet and other training materials will be adapted to include enhanced components of iCCM. CLHWs will be trained to identify lower chest indrawing in children below 5 years, to recognize and record danger signs of illness as described in the WHO-UNICEF training package for CLHWs, and refer or take any child with a danger sign to a health facility.^27^ All CLHWs, study supervisors, IOAs, and study coordinators will be trained in study objectives and strategy, and iCCM modules.

The CLHWs training materials will be translated into respective local languages. Government trainers will be involved in the training of the CLHWs. The CLHWs and supervisors of intervention clusters and IOAs will be trained in enhanced iCCM, whereas the CLHWs and supervisors from control clusters will be trained in standard iCCM. The training modules will include 6-day scheduled classroom and clinical training sessions. All CLHWs will be evaluated post training and those found weak will be retrained.

Additionally, all study staff will be trained in Good Clinical Practices (GCP). The study sites will also develop a system of periodic refresher training of CLHWs, supervisors and IOAs through video demonstration and/or clinical practice including pulse oximetry.

Standardization of measurement of respiratory rate, pulse oximetry, body temperature, MUAC and identification of danger signs will be done periodically (at least once every six months) for all CLHWs, supervisors and IOAs. The study physicians at every site will conduct these standardizations.

#### Study oversight, supervision and monitoring

A study supervisor will independently validate all children assessed as potential enrollees by a CLHW in both intervention and control groups. Supervisors will confirm pulse oximetry findings by CLHWs in the intervention group. A proportion of the follow-up visits will be accompanied by the supervisors, who will also conduct independent unaccompanied visits. Principal and co-investigators, study coordinators, and managers will also visit study sites to review project quality and cross-check a small proportion of collected CRFs and provide feedback as required.

Periodic meetings will be held to review research team performance, enrolment progress, field visit observations, address problems and identify solutions. Government managers at district/sub-district level will be involved in supervision and monitoring activities.

### External monitoring

WHO study coordinating team will visit each site prior to initiation of implementation to assist in preparation and planning. All sites will submit monthly progress report to WHO. WHO monitors will visit each site periodically to assess progress, compliance with study protocols, quality and accuracy of data collected, quality of care and child safety. In those visits a proportion of completed CRFs and all serious adverse event (SAE) reporting forms will be reviewed. Recommendations from the monitoring visits observations will be discussed with the research teams and follow up actions will be undertaken.

Data-based monitoring will be carried out by the central data coordination centre (DCC) in collaboration with the WHO study coordinator.

### Logistics and commodities

Trained CLHWs will be equipped with necessary medicines, equipment and logistics for pneumonia case management such as oral amoxicillin, pulse oximeter, thermometer, weighing scale, respiratory rate counting timer, MUAC tape, CRFs and other necessary materials. The study teams will monitor the CLHW’s equipment for periodic standardization and replacement of faulty devices, if any. The consumables will be replenished as required.

### Data collection instruments and procedures

CLHWs, study supervisors and IOAs will collect data. The supervisor will complete a confirmation form for each case before enrolment. A follow-up form (only in the intervention group) and pulse oximeter use checklist will also be completed for each case separately by the CLHWs and supervisors. The IOAs will complete an outcome assessment form on days 6 and 14 for both intervention and control groups. IOAs will fill a separate form describing for SAE. Completed forms will be reviewed by supervisors for completeness and corrected before data entry. Supervisors and IOAs will submit CRFs to the study site office. A coordinator/co-investigator will check the CRF to evaluate their completeness. Queries will be sent back to the field before CRFs are transferred to the data entry teams.

### Data management

The data management team at each site will be responsible for the site’s day-to-day data management activities. The DCC, an independent institution will coordinate all study data. DCC will develop a common database capable of keeping automated record of all changes, will be used by all sites. A DCC team will help to set up data management systems at all sites and train the local data managers and data entry operators. All sites will be periodically upload data at the central DCC server after application of range and across form consistency checks. DCC will run checks on uploaded data and the queries will be sent to the site-specific data managers for resolution. After resolution of these queries, the clean database will be uploaded to the DCC server site.

### Data analysis

The primary analysis will be a per-protocol combined analysis across all the sites. Simple comparisons of means and proportions by treatment groups will be used to check whether the randomization scheme resulted in baseline comparability. The analyses will compare non-inferiority between the standard iCCM and the enhanced iCCM. The comparison of proportions of children with treatment failure in each treatment group and the difference in the risk of treatment failure together with 95% confidence interval will be calculated. Data adjusted for any covariates which may be unbalanced at baseline and likely to influence the primary outcomes. Those who will be lost to follow-up or withdraw consent after enrolment will not be included in the analysis. An intention-to-treat analysis will also be conducted. Secondary analyses will be performed to investigate the secondary outcomes. Additionally, univariate and multi-variate regression analyses will be performed to identify predictors of treatment failure.

Separate data analysis workshops will be undertaken at the end of in the study for Asian and African sites. Data cleaning for the analysis of all the primary and secondary outcomes will be completed before the workshops and the databases will be locked.

### Flow of participants

**Figure 2:**
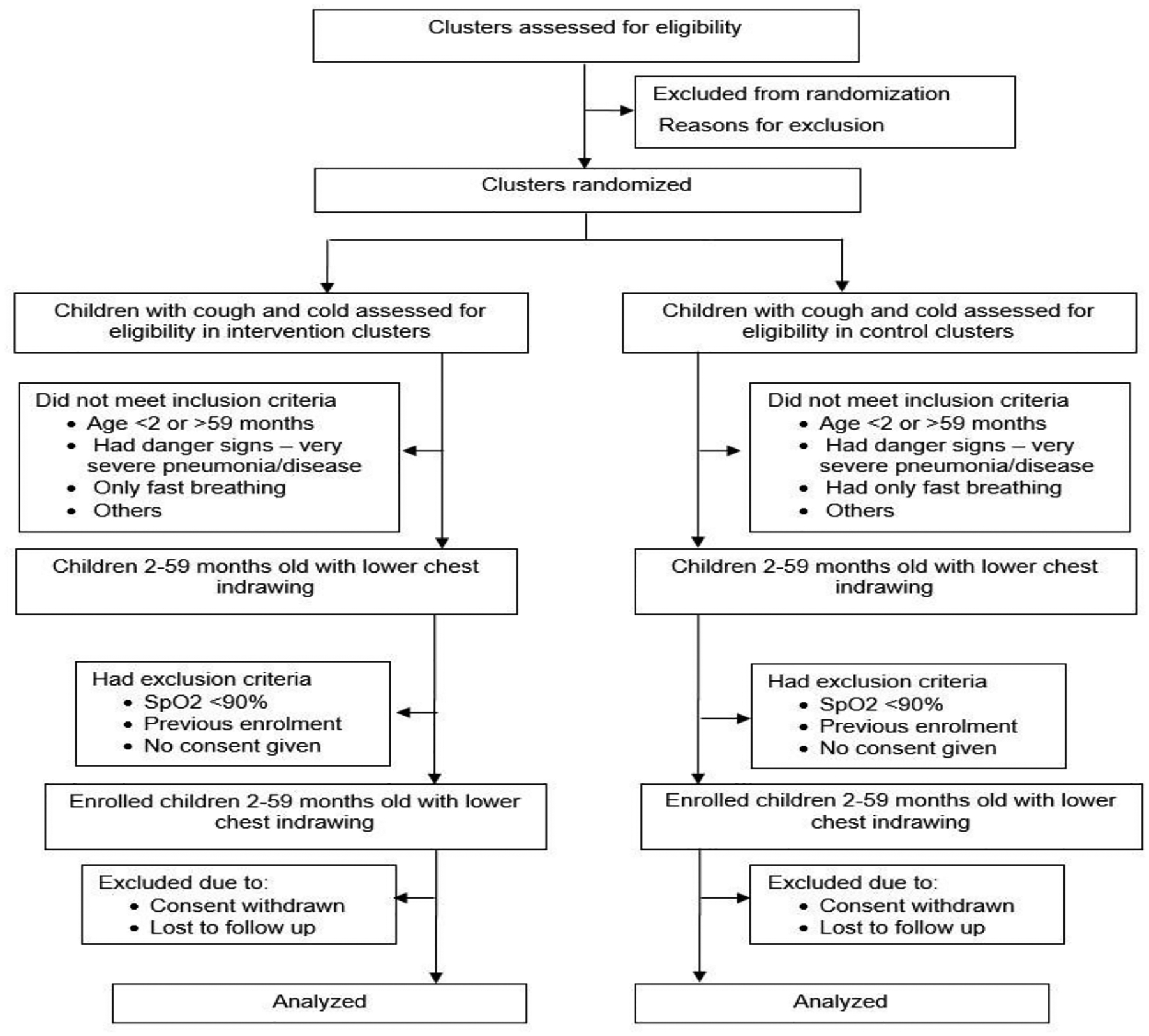
Flow of participants or trial profile.

The flow and number of clusters randomized and the participants through assessment of eligibility, follow-up, outcome ascertainment and analysis will be presented in Figure 2, along with exclusions and withdrawals at all timepoints.

### Data safety monitoring board

WHO will constitute an independent data safety monitoring board (DSMB) consisting of clinician, researcher and a statistician to review the progress of the trial at regular intervals and assess safety of the intervention. The DSMB will advise the team on study continuation, modification or termination on pre-decided stopping rules. In addition to the local ethics committee, all deaths and SAEs in enrolled participants will be reported to WHO for further communication to the DSMB.

### Serious adverse events

In case a child presents with a serious adverse events (SAEs) such as death, anaphylactic reaction, diarrhoea with severe dehydration, disseminated or severe rash from antibiotic/oral amoxicillin, the CLHW will contact the study supervisor and IOA. The IOA will confirm and document the SAE and report to the site’s study coordinator or investigator. All SAEs will be reported to WHO within 48 hours of the occurrence.

### Ethical considerations

Ethical clearances will be obtained from all site ethical review committees and WHO. Additional national, regional or state approvals will be obtained wherever required. The safety of enrolled children in this trial will be ensured by close monitoring and follow-up. CLHWs will be trained to facilitate referral. The trial will follow Council for International Organizations of Medical Sciences and GCP guidelines. Study communities in both intervention and control clusters will be informed through community representatives. A written individual informed consent in local language will be obtained from the parent or caregiver of the participant prior to enrolment. The consent form will be read out for illiterate parents and a thumb imprint will be taken witnessed by an impartial literate witness.

### Confidentiality and data handling

A unique participant identification (ID) number will be given to each participant on enrolment. The consent form, CRFs and any other forms linking participant personal information to study ID number will be kept in securely locked filing cabinets. The key linking participant name and ID will be kept confidential in a secured location at the site specific principal investigator’s office at each site with limited access. Proper documentation and storage of the metadata and any files or protocols relevant to data management will be handled with utmost care. Regular backups of the existing data will be done in appropriate intervals. All computers being used in the study will be password protected. None of the participants’ names or identifiers will be used in any publications or discussions regarding the study. The data/records will be kept until its use in any form including secondary analyses.

### Patient safety

The study procedures and data collection will not pose any significant physical, psychological, social, legal or other kind of risks to the participants. The clinical assessment, referral advice and illness management are routinely used in clinical practice. Every enrolled subject will be followed up till completion of treatment and assessed on day 14 of the enrolment. Pulse oximetry will be used to identify high risk hypoxaemic cases. Treatment failures will receive appropriate change of antibiotic therapy from the referral health facility according to standard clinical practice and those who need hospitalization will be referred to nearby public-sector hospitals immediately for appropriate management. Other risks to caregivers might include longer waiting time because of data collection and using pulse oximetry. However, the procedures will be explained in detail and any queries by parents/caregivers will be answered. The mobile phone number of the CLHW and study supervisors will be provided to parents/caregivers so that they could reach them if needed.

### Dissemination of results

Dissemination meetings will be organized in each country to share results and discuss their implications for the country. Country-level stakeholders including ministry of health programme managers, district and sub-district health managers, community leaders or representatives and academia will be invited. Results will also be disseminated at a regional and global level. Publication in national journals and presentation in national and international meetings will be encouraged.

## DISCUSSION

A significant reduction in pneumonia mortality can be achieved by improving access to appropriate diagnosis and standardized treatment. This study will assess the effectiveness and safety of CLHWs treating chest indrawing pneumonia in 2–59 months old children with oral amoxicillin. If found successful, it can increase the access to pneumonia treatment in low resource settings where referral to a hospital is not feasible for many families. The study will also collect evidence whether trained and supervised CLHWs are able to use pulse oximetry to screen hypoxaemia.

Our study has several strengths. First, the large sample size and multi-centre implementation will generate evidence that can be generalizable to Africa, South Asia and other similar settings. Second, standardized training, supervision, oversight, and monitoring will ensure quality, consistency, harmonized study procedures and implementation. Training and experience of implementing the study will increase knowledge, skill and confidence of CLHWs for better management of children with pneumonia. Third, there will be rigorous internal and external monitoring. Fourth, CLHWs in the intervention group will use pulse oximetry to identify and refer hypoxaemic children. Hypoxaemia is a key predictor of adverse outcome in pneumonia cases and difficult to identify clinically.^28–32^ Fifth, community mobilization and IEC activities will increase awareness about services provided by the CLHWs and generate demand for care within communities. The study will also help identify the most potent strategies to increase parents’ or caregivers’ knowledge of symptoms of childhood illnesses and improve health care-seeking behaviours.

Study has some limitations. First, enrolled children will be clinically diagnosed using iCCM protocol, without any microbiological and/or radiological methods. They are not available at community level in resource-limited settings where iCCM is recommended. Second, blinding of intervention therapy is not possible. Third, there may be an element of subjectivity involved in the assessment of treatment failure based on the presence or absence of clinical signs. To reduce bias, IOAs who are blinded to the treatment regimen will conduct outcome assessment both intervention and control groups.

## CONCLUSION

The results from this study are expected to contribute to the evidence base for potential revision of WHO/UNICEF iCCM protocol, its harmonization with the IMCI tool. Lessons learned during implementation will help to accelerate subsequent program scale-up.

## Figures and Tables

**Table 1:** Study site characteristics.

Country	Bangladesh	India	Ethiopia	Malawi
**Geographical location**	6 sub-districts in Barisal district under Barisal division: 176 community clinics	Palwal district, Haryana State: 92 sub-centers	4 highland districts: 92 health posts attached to 20 health centers	2 districts: 44 health facilities in 248 village clinics
**Population**	~1.1 million	~ 1.2 million	~ 0.5 million	~ 0.5 million
**Estimated number of under five year old children**	110,000	120,000	65,000	81,000
**Characteristics of clusters**				
Name	Union	Health sub-centers	Health center	Health center
Median population	25492	10318	25492	20926
**Characteristics of the CLHWs**				
Designation	CHCP	ASHA	HEW	HSA
Number of CLHWs	176	876	198	289
Basic education	12^th^ grade	Literate/8^th^ grade	10^th^ grade	12^th^ grade
Training	12 weeks training on primary health care	42 weeks training of ASHA training modules^33^	12 months training in hygiene, sanitation, disease prevention and family health	3 months training in health promotion and prevention
Place of work	Community clinic	Households	Health post	Village clinics
Population covered	6000–7000	1000	1500–2500	1600–1800

CHCP: Community health care provider, ASHA: Accredited social health activist; HEW: Health extension worker; HSA: Health surveillance assistant.

**Table 2:** Management of pneumonia in the intervention and control groups.

Intervention group CLHWs trained, supplied and supervised to: treat chest indrawing pneumonia in 2–59 month old children with oral amoxicillin for 5 days refer children with danger signs to hospital (after first dose antibiotic) Conduct pulse oximetry on children with pneumonia and refer hypoxaemic children to hospital which will be confirmed by supervisors Treat fast breathing pneumonia in children 2–59 months of age as part of iCCM protocol	Control group CLHWs continue to: refer all 2–59 month old children with chest indrawing pneumonia (after first dose of antibiotic) refer children with danger signs (after first dose of antibiotic) T reat fast breathing pneumonia in children 2–59 months of age as part of iCCM protocol Study supervisor to perform pulse oximetry (not part of treatment strategy)

**Table 3:** Implementation strategy.

Activity	What	Who	When	Where	How
**Intervention clusters**					
Case identification	Detection of sick children aged 2–59 months with cough or difficulty in breathing	CLHWs	Child with an illness is seen by the CLHW either at their place of work or during routine home visits	Community clinic in Bangladesh, Health Post in Ethiopia, Household in India and Malawi	Clinical assessment
Screening and enrolment	Assessing child with cough or difficulty in breathing for presence of chest indrawing and eligibility for enrolment	CLHWs	Chest indrawing is detected in a child (aged 2–59 months) with cough or difficulty in breathing.	Community clinic in Bangladesh, Health Post in Ethiopia, Household in India and Malawi	Clinical assessment, pulse oximetry and consent
Confirmation of cases	Validation of eligible cases identified by CLHW	Study Supervisor	An eligible child is identified by a CLHW and supervisor is informed	Community clinic in Bangladesh, Health Post in Ethiopia, Household in India and Malawi	Clinical assessment, pulse oximetry and confirmation of CLHWs findings
Treatment provision	Oral amoxicillin for 5 days	CLHWs and caregivers	After enrolment and consent	First dose given at place of enrolment and remaining doses given at home	Practical demonstration of giving the first dose in front of mother/caregiver
Follow up	Follow up assessments of children under intervention treatment	CLHWs	On days 2,4 and 7 after enrolment	Community clinic in Bangladesh, Health Post in Ethiopia, Household in India and Malawi	Clinical assessment, pulse oximetry and checking treatment adherence
Supervision	Assessing subsample of enrolled cases on follow-up days for quality assurance	Study Supervisor	Follow-up visits on days 2, 4, and 7	Community clinic in Bangladesh, Health Post in Ethiopia, Household in India and Malawi	Confirmational assessment
Outcome assessment	Assessing study outcomes for all enrolled cases	Independent Outcome Assessor	Days 6, 14 and if patient deteriorated in between	Patients household or a hospital if patient was admitted	Clinical assessment and filling outcome assessment CRF
**Control clusters**					
Case identification	Detection of cases	CLHWs	Child with an illness is seen by the CLHW either at their place of work or during routine home visits	Community clinic in Bangladesh, Health Post in Ethiopia, Household in India and Malawi	Clinical assessment
Screening and enrolment	Detection of eligible cases	CLHWs	Child is seen by the CLHW either at their place of work or during routine home visits	Community clinic in Bangladesh, Health Post in Ethiopia, Household in India and Malawi	Clinical assessment, and consent
Confirmation of cases	Validation of eligible cases identified by CLHW	Study Supervisor	An eligible child is identified by a CLHW and supervisor is informed	Community clinic in Bangladesh, Health Post in Ethiopia, Household in India and Malawi	Clinical assessment, and confirmation of CLHWs findings, pulse oximetry
Treatment provision	Refer to a referral facility	CLHWs	After enrolment and consent	First dose given at place of enrolment and refer to a referral facility	Counselling for referral
Follow Up	NA	NA	NA	NA	NA
Supervision	NA	NA	NA	NA	NA
Outcome assessment	Outcome assessment	Independent Outcome Assessor	Days 6, 14 and if patient deteriorated in between	Patients household or a hospital if patient was admitted	Clinical assessment and filling outcome assessment form

NA: Not applicable
